# Myositis/myasthenia gravis caused by immune checkpoint inhibition: A report of two cases and a brief review of the literature

**DOI:** 10.3892/mi.2024.210

**Published:** 2024-12-03

**Authors:** Zenia Elavia, Victoria Jimenez, Roxanne Lockhart, Allison Muha, Mohamed Kazamel

**Affiliations:** Department of Neurology, University of Alabama, Birmingham, AL 35294, USA

**Keywords:** cemiplimab, immune checkpoint inhibitors, immune-related adverse events, intravenous immunoglobulin, myasthenia gravis, myositis, nivolumab

## Abstract

Immune checkpoint inhibitors (ICIs) have revolutionized cancer therapy by enhancing the ability of the immune system to combat malignancies. Nivolumab and cemiplimab, monoclonal antibodies targeting programmed cell death protein 1, have exhibited notable therapeutic efficacy; however, they are associated with immune-related adverse events (irAEs). The present study describes the cases of 2 patients, a 71-year-old male with metastatic esophageal adenocarcinoma and a 66-year-old female with metastatic squamous cell carcinoma who developed acute/subacute onset rapidly progressive myositis/myasthenia gravis (MG) following treatment with nivolumab and cemiplimab. Both patients had negative MG antibody panels and the first had uninformative electrodiagnostic testing, rendering the diagnosis challenging. Additionally, 1 patient had myocarditis and the other had hepatitis. The mechanisms of ICI-induced myositis/MG remain unclear. Early recognition and intervention are vital for the prevention of severe morbidity and mortality. Treatment strategies, including the termination of the offending ICI medication, steroids, intravenous immunoglobulin (IVIG) and plasma exchange, should be tailored based on individual patient responses, and physicians should not hesitate to commence IVIG or plasma exchange once the diagnosis is established.. These cases underscore the importance of vigilance for myositis/MG in patients treated with ICIs, even with uninformative serological and electrodiagnostic testing, and the need for collaboration between multiple disciplines in managing complex irAEs including the associated myocarditis and hepatitis.

## Introduction

Immune checkpoint inhibitors (ICIs) have emerged as critical tools in the treatment of various malignancies by harnessing the potential of the immune system to combat cancer cells (1,2), transforming cancer treatment in the era of precision medicine (2). FDA-approved drugs, such as cemiplimab, ipilimumab, nivolumab and pembrolizumab, targeting cytotoxic T-lymphocyte-associated protein 4 (CTLA-4) and programmed cell death protein 1 (PD-1) checkpoints, have had a profound effect on patients with various types of cancer, including melanoma, non-small cell lung cancer, renal cell cancer and classical Hodgkin lymphoma (3,4). Nivolumab and cemiplimab, monoclonal antibodies targeting PD-1, are notable ICIs that have demonstrated notable therapeutic efficacy. However, the use of ICIs is associated with the risk of developing immune-related adverse events (irAEs), which can affect diverse organ systems. These potentially include conditions, such as diabetes, hypothyroidism, adrenal insufficiency, interstitial pneumonia, colitis, renal impairment and liver dysfunction (5). Neurological adverse effects are rare, occurring in <3% of patients treated with ICIs, including axonal polyneuropathies, Guillain-Barré syndrome, myositis, myasthenia gravis (MG), posterior reversible encephalopathy syndrome, aseptic meningitis, enteric neuropathy, transverse myelitis, and autoimmune encephalitis (5,6). MG, an autoimmune neuromuscular junction transmission disorder characterized by fatigable muscle weakness, is a commonly reported irAE associated with ICI therapy (1). Nivolumab and cemiplimab-induced myositis/MG presents a clinical challenge due to its rarity and the need for prompt recognition and management to prevent life-threatening outcomes (2).

The present study describes the cases of 2 patients who developed myositis/MG following nivolumab and cemiplimab therapy, a 71-year-old male and a 66-year-old female. In addition, a brief review of the literature was performed. The present study aims to shed light on the clinical presentation, diagnostic-associated difficulties and therapeutic interventions encountered with this unique clinical condition.

## Case report

### Case 1

**Figure 1:**
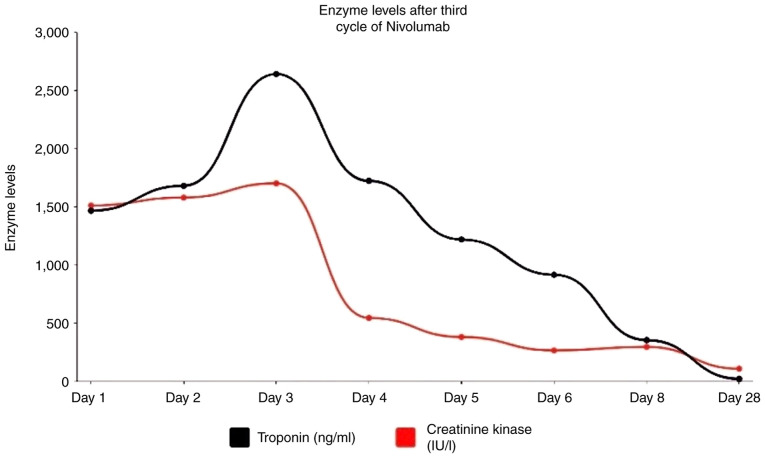
Temporal profile of creatinine kinase and cardiac troponins levels for patient 1.

Case 1 involves a 71-year-old male with a previous medical history of hypertension, hyperlipidemia, obstructive sleep apnea, acid reflux disease and esophageal adenocarcinoma that had metastasized to the liver. He received a treatment regimen consisting of leucovorin, oxaliplatin and 5-fluorouracil (FOLFOX), along with nivolumab and radiotherapy, to address the esophageal cancer. At 5 weeks after commencing treatment with nivolumab, he presented to the authors' oncology service at the University of Alabama (UAB; Birmingham, AL, USA) with chief complaints of acute onset double vision, fatigable weakness and shortness of breath. His social history entailed the consumption of 1 beer weekly. He had no history of illicit drug abuse. He had quit smoking 33 years prior. His family history was positive for colon cancer in an aunt on his father's side of the family. His father had a history of heart valve disease and ischemic stroke. His mother had a history of coronary artery disease. He was empirically treated with 100 mg prednisone for elevated hepatic transaminases. Upon a physical examination, his vital signs were found to be stable and his oxygen saturation was 98% in room air. Upon a neurological examination, his speech was clear with good naming and repetition. A cranial nerve examination revealed right eye ptosis, impaired extraocular muscle movements in all directions, and proximal more than distal limb weakness. Deep tendon reflexes were preserved all over with down-going toes. An examination of his sensory and cerebellar functions did not reveal any notable findings. A further evaluation revealed elevated troponin levels peaking at 2,641 ng/ml (Fig. 1) with electrocardiogram changes suggestive of non-ST-elevated myocardial infarction (NSTEMI) and creatine kinase (CK) at 1,511 IU/l (Fig. 1). He was admitted to the hospital, and cardiology was consulted for his NSTEMI.

The new and acute onset symptoms during chemotherapy made it more likely to be immunotherapy-induced myositis/MG rather than a steroid induced myopathy case. The neurology and rheumatology teams were consulted thereafter due to a concern for ICI-induced myositis/MG. His Myasthenia Gravis Foundation of America (MGFA) classification was deemed to be class IIIa. His laboratory test results are listed in Table I. A nerve conduction study (NCS) and an electromyography (EMG) revealed evidence of mild axonal sensory and motor neuropathy with no evidence of myopathy. Repetitive nerve stimulation (RNS) testing did not reveal any decrement or facilitation response. A brain MRI with contrast did not reveal any notable findings or acute changes and did not reveal any brain metastases. The patient was commenced on high-dose prednisone at 1 g/kg/day for 3 days and then transitioned to prednisone at 2 mg/kg/day on day 3, and subsequently to methylprednisolone at 1,000 mg daily for 3 days due to a concern for immunotherapy-induced cardiotoxicity. The patient was also commenced on intravenous immunoglobulin (IVIG) as per the neurological recommendation due to the concern for generalized MG and/or myositis, despite negative EMG and serological testing. The condition of the patient clinically improved with decreased levels CK of 295 U/dl and troponin upon discharge (Fig. 1). He was discharged on 1 mg/kg/day (100 mg) of prednisone daily until his follow-up appointment with his oncologist, following which prednisone was tapered slowly over a period of 6 to 8 weeks.

After 2 weeks, he presented again to the UAB Medical Center for the third time in 2 months with worsening symptoms, including drooping of the right eyelid, double vision, shallow breathing, difficulty swallowing, proximal limb weakness, dysarthria and failure to thrive. A neurological examination revealed right eye ptosis, nasal speech, impaired extraocular muscle movement and fatigable proximal muscle weakness. These clinical findings raised a strong suspicion of an ongoing myasthenic process, particularly in the setting of continual decreasing levels of CK (98 U/dl). His hospital course was complicated, and his condition rapidly deteriorated. He was placed on intermittent bi-level-positive airway pressure support to improve his breathing effort (now MGFA class IVb, 8 weeks from the onset of his myositis/MG). He was commenced on plasmapheresis. However, the clinical condition of the patient only minimally improved in terms of truncal strength, swallowing and diaphragmatic weakness. The palliative care team engaged with the patient and family to address the goals of care. The patient and family desired to forgo further chemotherapy and return home with hospice care to maximize the quality of life in the remaining time. The patient passed away 1 month thereafter.

### Case 2

Case 2 involves a 66-year-old female patient with a previous medical history of hypertension and hyperlipidemia who was commenced on cemiplimab therapy for metastatic squamous cell carcinoma at an outside cancer clinic. She received her first dose and within 10 days; she had generalized weakness, difficulty rising from a chair and walking with an unsteady gait. Over the following 2 weeks, she began to suffer from weakness in the proximal upper limbs, swallowing difficulties, dysarthria, diplopia and right eye ptosis. Her clinical image was consistent with MG, although tests for acetylcholine receptor (AChR) and muscle-specific kinase (MuSK) antibodies yielded negative results. She was empirically commenced on an immediate course of prednisone and pyridostigmine, although the latter was terminated due to diarrhea. Additionally, the patient received five doses of IVIG, without exhibiting a notable improvement in her symptoms. She was then referred to the UAB Medical Center for oncologic and neurologic management at 2 months from the initial dose of cemiplimab. Of note, she did not have any history of smoking, alcohol consumption, or illicit drug abuse. Her family history was negative for cancer or neurological disorders.

Upon an initial examination, she endorsed continued generalized weakness, a 28-pound weight loss due to dysphagia, voice changes and a difficulty with deep breathing. Her only therapeutic at the time was prednisone which had been tapered from 60 to 20 mg. A neurological examination revealed ptosis and a facial droop worse on the right side compared with the left side, 4/5 strength in the majority of the proximal muscle groups, normal reflexes, and intact all-modalities sensory examination (MGFA class IIIb, 7 weeks from the onset of myositis/MG). The results of her laboratory are presented in Table I. Given her facial asymmetry, a head CT scan was obtained, which did not reveal any notable findings. A brain MRI and c-spine with and without contrast were also non-informative (other than moderate cervical stenosis). A NCS and EMG revealed electrophysiologic evidence of diffuse irritable myopathy. RNS did not reveal a decremental response, yet concentric needle jitter analysis revealed all six pairs with increased jitter and an elevated mean consecutive difference of 69.2 msec, as can be seen with myasthenia gravis.

The patient completed five sessions of plasma exchange therapy over 10 days of hospital stay at the UAB Medical Center. She exhibited a gradual improvement in her symptoms and although she initially failed a swallow test, she was able to slowly advance her diet along with mirtazapine therapy to improve her appetite. On an outpatient follow-up at 1 week after discharge, she exhibited an improvement in her gait and speech. The patient continued to have difficulty with dysphagia, diplopia, and proximal weakness and prednisone was increased to 40 mg. She was then set up for an initiation of monthly outpatient combined plasma exchange with IVIG 1 week later. The last time she was seen at the UAB Outpatient Kirklin Clinic was 3 months later. She still had bilateral mild ptosis, diplopia when looking upwards (after ~10 sec) and to the right, and 4/5 muscle strength in the bilateral external arm rotators, hip flexor and abductor muscles (MGFA class IIa).

## Discussion

The present study describes two cases of ICI-related myositis/MG. The first patient began to exhibit symptoms of myositis/MG 5 weeks after receiving nivolumab and progressed to MGFA class IV over a period of 8 weeks. The second case developed the disease only 10 days after taking one dose of cemiplimab and progressed to MGFA class III within 7 weeks. Both cases clearly reflect the aggressive nature of the disease compared to what is typically observed in autoimmune MG cases. In the first case, a poor outcome was observed with the commencement of steroid treatment, despite upgrading the treatment with IVIG and plasma exchange later. In the second case, a more proactive and sustained treatment approach, including IVIG and plasma exchange treatments followed by maintaining the same modalities on an outpatient basis, led to a more favorable outcome. In their study, Safa *et al* (7) reported that 63% of their 65 patients had a similar aggressive course developing moderate to severe muscle weakness (MGFA class III to V) following ICI with the vast majority of patients (96%) requiring hospitalization. The median time between ICI treatment initiation and the first MG symptom was 4 weeks (range, 6 days to 16 weeks) (7). Myositis was noted in more than one third of their patients and both of the patients in the present study developed serological or electrophysiological evidence of myositis. In the present study, the 1st patient developed myocarditis and the 2nd patient developed hepatitis, which were reported in 8 and 9% of the patients in the study by Safa *et al* (7), respectively. Another smaller cohort study demonstrated that 8 out of 10 reported patients developed the triad of MG, myositis and myocarditis (8). Typically, in regular cases of MG, classes IV/V are encountered in only 2-10% of cases and the time from onset to class IV/V status ranges between 2-3 years (9,10). Evidence of concurrent myositis exists in only 0.9% of typical MG cases (11).

Nivolumab and cemiplimab, the offending agents in both cases presented, are monoclonal antibodies that bind to PD-1 receptors, found on the surfaces of activated T-cells. They prevent the binding of tumor-secreted PD-1 ligands to the PD-1 receptor (4,12). Blocking the PD-1 receptor can enhance T-cell antitumor activity; however, this may also result in an increased CD8/CD4 ratio and reduced regulatory T-cell numbers (13). While ICIs have exhibited notable responses in a number of malignancies, they can cause irAEs (2). The exact mechanisms of irAEs are not yet fully understood; however, but they are c to stem from an imbalance between autoimmunity and immune tolerance. Immune checkpoints, such as PD-1 and CTLA-4 play a crucial role in maintaining self-tolerance and preventing autoimmunity. When these natural immune ‘brakes’ are released, this can lead to unchecked activated T-cells targeting self-antigens, the release of inflammatory cytokines, and ultimately, in inflammation and tissue damage, clinically presenting as autoimmune disorders (3,6). In a previous study, peripheral blood mononuclear cellular typing was performed before and after nivolumab treatment in a patient who developed MG/myositis/myocarditis, and revealed an increased expression of CD8 and cytolytic activity markers, whereas CD4 T-cell and T-regulatory cell activity were suppressed (14). An elevated CD8/CD4 ratio was reported in another patient with nivolumab-related MG/myositis (15). Other hypotheses have been proposed, including that the use of PD-1 inhibitors may lead to a pro-inflammatory cascade involving T-lymphocytes, interleukins (IL-2, IL-6, IL-17) and tumor necrosis factor-alpha, which may contribute to the development of MG. ICIs may also increase the availability of ligands for CD28, activating potentially self-reactive T-cells. Nivolumab could unmask latent autoimmunity toward the AChR that was not clinically manifest earlier (2). In the present study, case 1 also developed concurrent myocarditis after commencing treatment with PD-1 inhibitors. The pathophysiology of this overlap suggests that PD-1 expression on cardiomyocytes protects the myocardium during stress. When there is damage to the heart during ICI blockade, T-lymphocytes are less likely to attack cardiac antigens, and PD-1 is less likely to protect the heart (6,16).

In addition to the two largest case cohorts (7,8), herein, a review of the literature (Table II) (2,12,14,16-29) revealed 19 additional case reports published between 2014 and 2021. In their large cohorts, Safa *et al* (7) and Weaver *et al* (8) reported that ~1/3 of cases were seronegative when it came to MG antibodies. An additional five individual case reports revealed negative anti-AchR antibodies, all consistent with both of the cases described herein (12,23,26,27,29). Of note, 80-85% of individuals who suffer from *de novo* MG in general have AchR antibodies. In generalized MG patients who are seronegative for AChR antibodies, 50-70% test positive for MuSK antibodies. However, these antibodies are not always found in ICI-mediated MG, and when they are, they are much less common than in typical MG (6). Thus far, there are only four reported cases of anti-MuSK antibodies found in a patient with ICI-induced MG (7,8,16). This was linked to a very poor prognosis, resulting in severe respiratory muscle failure (7,8,16).

The overlap of MG with myositis creates a diagnostic challenge. NCS and EMG are often used for diagnosis; however, repetitive nerve stimulation testing is generally insensitive especially in ocular MG cases (6). It remains unclear whether elevated CK levels reflect a concurrent process or primary myositis predominantly involving the oculobulbar muscles (6). In the cases described herein, the fatigable component of weakness with the descending distribution favored a myasthenic process. However, the negative MG serologic and electrodiagnostic testing in case 1 in addition to the elevated levels of CK at time of onset favored a concomitant myopathic process.

### Treatment considerations

For ICI-associated MG, treatment typically involves discontinuing the offending ICI. Earlier reports have indicated that patients who receive IVIG or plasma exchange as the initial treatment have higher rates of symptom improvement compared to those treated with only steroids (6-8). IVIG or plasma exchange are more effective when used as the first-line treatment option, as some patients deteriorate despite the second-line use of IVIG or plasma exchange after initial steroid treatment has failed (2,7). This pattern was also noted in the cases in the present study. Fatality due to ICI-associated MG has been reported in 20-38% of cases (2,6,7). Complete symptom resolution is relatively rare, and prognosis is guarded. In the first case described herein, the patient was discharged to hospice care with outpatient follow-up with a neurologist. Current recommendations indicate a trial of pyridostigmine with or without concurrent steroid therapy for patients with MGFA class I (ocular symptoms only) or class II (mild generalized weakness). A re-challenge with ICI can be considered in patients with MGFA class 1 or 2, as reported in two exceptional cases in the literature (6).

### Clinical implications and challenges

Prior to initiating ICIs, it is crucial to screen patients for autoimmune diseases and analyze CK levels. Patients should be informed about the risk of developing irAEs (rheumatologic and neurologic), despite their low incidence rates. Based on the present case reports and previous data, a closely collaborating team comprising neurologists, rheumatologists and oncologists is necessary to diagnose and manage life-threatening adverse effects promptly and effectively (2,6). The cases described herein also underscore the importance of not relying solely on serological markers or electrodiagnostic testing, as this can lead to diagnostic delays with a potential impact on patient outcomes.

### Limitations

The present case reports have limitations, including a lack of comprehensive understanding of the underlying pathophysiology of ICI-induced myositis/MG and the absence of standardized diagnostic criteria. Single-fiber EMG was not performed on the first patient as it was deemed to be technically challenging as the patient spent most of his hospital course in the ICU or the stepdown unit.

### Conclusion

ICI medications have revolutionized cancer therapy. However, they are associated with the risk of developing irAEs, including myositis/MG, which present a unique set of diagnostic and management challenges. The causes of ICI-induced myositis/MG remain unclear and preclinical studies are required to elucidate the pathophysiology of these irAEs. There are several diagnostic challenges, including that this condition is rare and sometimes the serological markers and electrodiagnostic testing are both negative. The potentially associated myocarditis and hepatitis may render the diagnosis even more challenging and require a team from multiple disciplines to take care of such cases. Prompt recognition and intervention are essential in preventing severe morbidity and mortality in these patients, since the disease appears to be more aggressive than what is observed in typical MG cases. When someone has ICI-induced MG, they are usually treated by terminating the ICI that is causing it, commencing treatment with corticosteroids, and early treatment with IVIG and/or plasma exchange. Prospective longitudinal studies enrolling larger number of patients could define the true incidence of myositis/MG after ICI and differentiate those who had a subclinical autoimmunity that manifests only following exposure to ICI from new-onset disease. They could also better answer the question whether it is safe to reintroduce ICI treatment in a patient who was treated earlier from ICI induced myositis/MG, since the current safety data are all derived from case reports and small cohort studies. The two cases described herein underscore the importance of proactive approaches to monitoring and managing myositis/MG in ICI-treated patients, regardless of negative serological markers and electrodiagnostic testing. As the use of ICIs continues to expand in cancer therapy, healthcare providers need to be vigilant and well-informed about the potential of these multisystemic irAEs and be prepared to provide early and specific interventions to optimize patient outcomes.

## Figures and Tables

**Table I:** Laboratory test results for patients 1 and 2.

Laboratory test	Patient 1	Patient 2
Serum electrolytes	Normal	Normal
Liver function tests	Low albumen (3.1 g/dl)	Low albumen (3.4 g/dl) and mildly elevated ALT (58 U/l) and AST (42 U/l)
CK	Shown in Fig. 1	Normal (55-69 U/l)
Differential blood count	Iron deficiency anemia and neutrophilia^a^	Neutrophilia^a^
Thyroid function tests	Normal	Normal
SPEP and IFE	Normal	Normal
Urine IFE	Normal	Normal
AChR antibody	Negative	Negative
MuSK antibody	Negative	Negative
VGCC antibody	Negative	Negative
Serum myositis panel	Negative	Not tested
Serum paraneoplastic panel	Negative	Positive low titer VGKC antibody (0.05 nMol/l)
Cerebrospinal fluid		
WBCs	0	Not tested
Glucose	Elevated (78 mg/dl)	
Protein	Elevated (65 mg/dl)	
Paraneoplastic panel	Negative	

^a^Likely steroid-induced. AchR, acetylcholine receptor; CK, creatine kinase; IFE, Immuonofixation; MuSK, muscle-specific kinase; SPEP, serum protein electrophoresis; VGCC, voltage gated calcium channel; VGKC, voltage gated potassium channel; WBCs, white blood cells.

**Table II:** Reported cases of ICI-induced myositis/myasthenia gravis in the literature.

Year of publication	Authors	Age/sex	Cancer	ICI used	Onset time	AchR antibody	CK (IU/l)	Clinical features	Electrodiagnostic Testing	Treatment	Outcome	(Refs.)
2014	Liao *et al*	70/F	Melanoma	Ipilimumab	2nd cycle	Positive	1200	Fatigue	Demyelinating polyneuropathy	Steroids, pyridostigmine, PLEX, and IVIG	Improved	(17)
2015	Johnson *et al*	69/F	Melanoma	Ipilimumab	3rd cycle	Positive	No data	Diplopia, ptosis, and dysphagia to solid foods	Decremental response to 3Hz stimulation.	Steroids, pyridostigmine, and PLEX	Improved	(18)
2015	Johnson *et al*	73/F	Melanoma	Ipilimumab	2nd cycle	Positive	No data	Dyspnea, proximal limb weakness		Steroids and pyridostigmine	Mortality (cancer)	(18)
2015	Loochtan *et al*	70/M	SCLC	Ipilimumab + nivolumab	16 days	Positive	No data	Dyspnea and generalized weakness	Decremental response to 3Hz stimulation	Steroids, PLEX, and IVIG	Death (hospice)	(19)
2016	Kimura *et al*	80/M	Melanoma	Nivolumab	1st cycle	Positive before and after ICI	7740	Dyspnea and muscle weakness	Data unavailable	Steroids, PLEX, and IVIG	Improved	(14)
2016	Maeda *et al*	79/M	Melanoma	Nivolumab	3rd cycle	Positive	1627	Diplopia and facial weakness	Data unavailable	None	Improved	(20)
2016	Sciacca *et al*	81/M	NSCLC	Nivolumab	3rd cycle	Positive	No data	Dyspnea	RNS negative. Single fiber electromyography: abnormal	Steroids	Improved	(21)
2016	Shirai *et al*	81/F	Melanoma	Nivolumab	22 days	Positive before and after ICI	8729	Dyspnea	RNS negative	Denied any treatment	Mortality (MG crisis)	(22)
2016	Polat *et al*	65/M	NSCLC	Nivolumab	3rd cycle	Negative	No data	Weakness	Patient declined EMG	Pyridostigmine	Improved	(23)
2017	Chang *et al*	75/M	Bladder cancer	Nivolumab	2nd cycle	Positive	1587	Dyspnea and weakness	Decremental response to 3Hz stimulation.	Pyridostigmine and IVIG	Mortality (cancer)	(24)
2017	Chen *et al*	57/M	NSCLC	Ipilimumab + Nivolumab	2nd cycle	Positive before and after ICI	2682	Ptosis, Dyspnea, and weakness	Active denervation and myopathic changes. No decremental response	Steroid and pyridostigmine	Improved	(25)
2018	Kang *et al*	75/M	Oral cavity cancer	Nivolumab	3 weeks	Negative	No data	Fatigue and weakness	Patient declined EMG	Steroids	Mortality	(12)
2019	Fazel *et al*	82/M	Melanoma	Nivolumab	2nd cycle	Anti-MuSK positive only	Elevated (level not mentioned)	Ptosis and Dysarthria	Data unavailable	Steroid and pyridostigmine	Mortality	(16)
2020	Veccia *et al*	65/M	NSCLC	Nivolumab	4 weeks after 2nd cycle	Negative	Not mentioned	Diplopia and ptosis	Evidence of proximal myopathy	IVIG, Steroids, and pyridostigmine	Mortality	(26)
2020	Jeyakumar *et al*	86/M	SCC	Cemiplimab	3 weeks after 1st cycle	Negative	6407	Fatigue, decreased vision, aches	Not performed due to rapid decompensation	Steroids, PLEX, IVIG	Mortality	(27)
2021	Canino *et al*	90/F	SCC	Cemiplimab	5 weeks after 1st cycle	Positive	157	Facial, weakness bulbar symptoms, dysphagia, hypophonia	Single fiber EMG; Nonpathological alteration	IVIG, steroids, pyrixostigmine	Improved	(28)
2021	Canino *et al*	75/M	Melanoma	Nivolumab	Two weeks after 1st cycle	Positive	20	Ptosis	Single fiber EMG; pattern of neuromuscular junction alteration	Steroids, pyridostigmine	Improved	(28)
2021	Bawek *et al*	68/M	Melanoma	Nivolumab	3 weeks after 2nd dose	Negative	Elevated	Proximal muscle weakness, double vision, dysphagia, and ptosis	Data unavailable	Pyridostigmine and IVIG	Discharged on hospice	(29)
2021	Tahir *et al*	76/M	Esophageal cancer	Nivolumab	6 weeks after initial cycle	Positive	Normal	Generalized weakness and diplopia	Patient declined EMG	IVIG and PLEX	Hospice care	(2)

M, male; F, female; AchR, acetyl choline receptor antibody; CK, creatine kinase; EMG, electromyography; IVIG, intravenous immunoglobulin; MuSK, muscle specific kinase; MG, myasthenia gravis; NSCLC, non-squamous cell lung cancer; PLEX, plasma exchange; RCC, renal cell carcinoma; RNS, repetitive nerve stimulation; SCLC, squamous cell lung cancer; SCC, squamous cell carcinoma.

## Data Availability

The datasets used and/or analyzed during the current study are available from the corresponding author on reasonable request.
